# Multigenetic pharmacogenomics–guided treatment shows greater improvements on motor symptoms compared to usual therapy in Parkinson’s disease: a small real-word prospective cohort study

**DOI:** 10.3389/fphar.2025.1502379

**Published:** 2025-03-25

**Authors:** Yifan Li, Mao Li, Miao Wang, Jiarui Yao, Fengzhu Li, Siyu Chen, Xi Yin, Zhongbao Gao

**Affiliations:** ^1^ Geriatric Neurological Department of the Second Medical Center and National Clinical Research Center for Geriatric Diseases, Chinese PLA General Hospital, Beijing, China; ^2^ Department of Neurology of the First Medical Center, Chinese PLA General Hospital, Beijing, China

**Keywords:** Parkinson’s disease, pharmacogenomics, single nucleotide polymorphisms, drug efficacy, personalized medicine

## Abstract

**Background:**

Dopamine replacement therapy is a cornerstone of Parkinson’s disease treatment. In clinical practice, there is considerable variability in patients’ responses, tolerability, and safety regarding anti-parkinsonian medications, which is largely influenced by genetic polymorphisms in pharmacokinetic and pharmacodynamic genes. However, the application of multigenetic pharmacogenomics-guided treatment (MPGT) to optimize therapeutic outcomes in Parkinson’s disease (PD) remains under-explored. In this study, we conducted a prospective cohort investigation to evaluate the potential benefits of MPGT on motor symptoms in PD patients.

**Methods:**

A total of 28 patients with PD were followed for 4 weeks. Among them, 22 patients underwent multigenetic pharmacogenomic testing, with 13 receiving treatments based on the test results (MPGT group). The remaining 15 received standard care (TAU group). Baseline characteristics, as well as changes in Unified Parkinson’s Disease Rating Scale (UPDRS) III scores and sub-scores, were compared between the two groups. Associations between various single nucleotide polymorphisms (SNPs) and treatment outcomes were analyzed using generalized linear models.

**Results:**

At the 4-week follow-up, the MPGT group showed significantly greater reductions in UPDRS III total scores (p < 0.05) and limb sub-scores (p < 0.01) compared to the TAU group. These differences remained significant after adjusting for increases in levodopa equivalent daily dose (*p =* 0.011 and *p =* 0.002, respectively) and piribedil use (*p =* 0.006 and *p =* 0.004, respectively). Patients homozygous for the major allele of rs4984241 (AA vs. AG+GG, *p =* 0.003), rs4680 (GG vs. GA+AA, *p =* 0.013), rs1076560/rs2283265 (CC vs. AC+AA, *p =* 0.039) and rs622342 (AA vs. AC, *p =* 0.043) showed greater improvement in total UPDRS III, postural instability and gait difficulty (PIGD), rigidity and tremor scores, respectively, compared to those carrying at least one minor allele.

**Conclusion:**

MGPT demonstrates significant potential as a valuable tool for personalized treatment in PD patients. Additionally, we identified several SNPs associated with the responsiveness to chronic administration of multiple anti-parkinsonian drugs. However, to confirm these findings, well-designed studies with larger, well-characterized samples are necessary.

## 1 Introduction

Parkinson’s disease (PD) is the second most common neurodegenerative disorder after Alzheimer’s disease (AD), affecting approximately 6.1 million people worldwide. Its incidence rises significantly with age (Ascherio and Schwarzschild, 2016; GBD, 2016 Parkinson’s Disease Collaborators, 2018), imposing considerable burdens on patients, their caregivers, and society (GBD, 2016 Parkinson’s Disease Collaborators, 2018). Dopamine replacement therapy (DRT), which aims at restoring dopaminergic transmission in the nigrostriatal pathway, is a core component of Parkinson’s disease treatment. The improvement of motor symptoms primarily relies on DRT, which includes levodopa preparations, dopamine agonists (e.g., pramipexole, rotigotine, and ropinirole), monoamine oxidase-B inhibitors (e.g., selegiline, rasagiline, and zonisamide), and catechol-O-methyltransferase inhibitors (e.g., entacapone, opicapone, and tolcapone). Additional pharmacological options include anticholinergics (e.g., trihexyphenidyl, benztropine), amantadine, and others (Armstrong and Okun, 2020).

Most PD patients receive multiple classes of medications based on clinical evidence and appropriately tailored strategies to achieve complementary benefits, as well as, minimizing high doses and dose-related adverse events. However, responses to dopaminergic drugs can vary significantly between individuals, influenced partly by genetic factors. Recent pharmacogenomic studies have demonstrated that single nucleotide polymorphisms (SNPs) in genes involved in dopamine signaling pathways can predict PD risk (McGuire et al., 2011), treatment-related adverse effects (Arbouw et al., 2009; Rieck et al., 2018; Redensek et al., 2019; Michalowska et al., 2020; Yin et al., 2021; Soraya et al., 2022), or the effectiveness of specific medications (Liu et al., 2009; Becker et al., 2011; Masellis et al., 2016; Xu et al., 2017; Miller et al., 2018; Zhang et al., 2021). Despite these findings, it remains uncertain whether SNPs can guide clinicians in selecting the optimal treatment strategies for PD.

Commercial multigenetic pharmacogenomic tests have been developed to provide drug recommendations based on proprietary algorithms, which have been shown to outperform single-variant testing in predicting treatment outcomes (Altar et al., 2015). Multigenetic pharmacogenomics-guided treatment (MPGT) has shown promise in the management of psychiatric disorders (Greden et al., 2019; Papastergiou et al., 2021; Kang et al., 2023); however, its application in PD treatment is not well-established. In this study, we conducted a prospective cohort to evaluate the efficacy of MPGT in PD patients compared with treatment as usual (TAU), and further analyzed the effects of different SNPs on the chronic administration of anti-parkinsonian drugs.

## 2 Materials and methods

### 2.1 Participants

We consecutively enrolled 35 PD patients who were admitted to the Geriatric Neurological Department of Chinese PLA General Hospital between July 2023 and December 2023. PD was diagnosed according to the 2015 Movement Disorder Society clinical diagnostic criteria for Parkinson’s disease. Patients with atypical parkinsonism, such as progressive supranuclear palsy, multiple system atrophy, and secondary parkinsonism, were excluded. Additionally, three patients whose chief complaint was non-motor symptoms (e.g., postural hypotension, hallucinations) while their motor symptoms remained well-controlled, as well as one patient with mutations in monogenic PD-related causal genes, were also excluded. A follow-up assessment was performed on 28 patients who had undergone adjustments to their anti-parkinsonian medication at the end of week 4 following medication adjustment. Multigenetic pharmacogenomic tests were conducted on 22 patients, of whom 13 patients received treatment based on the pharmacogenomics report (MPGT group). The remaining 15 patients, including those who did not undergo pharmacogenomic testing or underwent the test after medication adjustments, received standard care (TAU group). Medication adjustments in both groups were made by the same experienced movement disorder specialist. The study was approved by the Ethics Committee of the Chinese PLA General Hospital, and all patients provided written informed consent.

### 2.2 Clinical assessments

Demographic data, including age, gender and disease duration, were collected. The evaluation of motor and non-motor symptoms was conducted in the “ON” state in all PD patients. Baseline assessments for all patients included the Movement Disorder Society-sponsored Revision of the Unified Parkinson’s disease Rating Scale (MDS-UPDRS I–IV), the Hoehn and Yahr (H-Y) stage, and the levodopa equivalent daily dose (LEDD). Cognitive function was assessed using the Mini-Mental State Examination (MMSE) and the Montreal Cognitive Assessment (MoCA, Beijing Version). Quality of life was evaluated with the Parkinson’s Disease Questionnaire-39 (PDQ-39). Disease duration was defined as the time from the onset of the first motor symptoms to the time of enrollment in the present study. UPDRS III was reassessed at the 4-week follow-up. Sub-scores for motor functions, derived from relevant MDS-UPDRS Ⅲ items, were calculated separately for the following components: rigidity (items 3.3a–e), tremor (items 3.15–3.18), postural instability and gait difficulty (PIGD) (items 3.9–3.13), and limb function (items 3.4–3.8). Treatment outcomes were measured by reductions in the MDS-UPDRS III score and its sub-scores at the end of week 4.

The dosage increases in levodopa equivalent daily dose, levodopa and benserazide, carbidopa-levodopa, entacapone, pramipexole, piribedil, amantadine, selegiline, and rasagiline were also calculated.

### 2.3 Multigenetic pharmacogenomic testing

In this study, we identified single-nucleotide variant loci of 18 alleles or variants across 12 genes that have been reported to be associated with the metabolism, efficacy, or adverse effects of anti-parkinsonian medications (Supplementary Table S1). Genomic DNA was isolated from buccal samples, and genotyping was performed by Conlight Medical Inc. (Shanghai, China) using the MassArray (MALDI-TOF MS) genotyping method. This method categorizes medications into three categories for each participant: (1) “use as directed,” indicating minimal or no gene-drug interactions, allowing physicians to prescribe the medication under standard circumstances; (2) “moderate gene-drug interaction,” suggesting that the medication should be used following evaluation; and (3) “significant gene-drug interaction,” indicating that the medication should be administered with blood concentration monitoring or alternatives should be considered. A total of 13 commonly used anti-parkinsonian medications were included in our study: levodopa, pramipexole, piribedil, ropinirole, selegiline, rasagiline, entacapone, tolcapone, amantadine, rotigotine, benzhexol, istradefylline, and zonisamide.

### 2.4 Statistical analysis

For the baseline characteristics analysis, variables were appropriately described as means and standard deviations (SDs), or medians with interquartile (IQR) ranges (IQRs; first and third quartiles). For normally distributed data, separate two-tailed ANOVAs were performed, while Mann-Whitney U tests were used for non-normally distributed data. Categorical variables were analyzed using *χ* ^2^2 tests or Fisher’s exact tests. Hardy-Weinberg equilibrium was assessed with *χ* ^2^ tests. Genetic associations with short-term efficacy were examined using various genetic models, including dominant (aa + Aa vs. AA), recessive (aa vs. Aa + AA), and additive (AA vs. Aa vs. aa) models. Adjustments for LEDD or piribedil increase were made using a generalized linear model. Statistical significance was set at a two-tailed α level of 0.05. All statistical analyses were conducted using SPSS Statistics version 26.0 (IBM). The figures were generated using R, version 4.3.1.

## 3 Results

### 3.1 Demographic and clinical characteristics

The demographic and clinical characteristics of the 28 PD patients were detailed in Table 1. The mean ages were 69.69 ± 7.65 (range, 55.0–81.0) years and 67.40 ± 6.14 (range, 54.0–76.0) years for the MPGT and TAU groups, respectively, with median disease durations of 4.87 ± 3.44 years (range, 1.0–13.0) and 3.72 ± 2.16 (0.5–8.0) years. There were no significant differences in demographic characteristics between the treatment groups at baseline. However, the dosage of piribedil increased significantly in the MPGT group compared to the TAU group (z = −2.082, *p* < 0.05), while dosage changes for other medications were not significantly different (Table 1).

**TABLE 1:** Comparing of baseline demographics and clinical characteristics, as well as changes in medication doses between the two groups.

Characteristics	MPGT (n = 13)	TAU (n = 15)	*χ* ^*2*^/t/z	*p*-value
Gender (Male/Female)	9/4	6/9	2.392	0.122
Age (years)	69.69 ± 7.65	67.40 ± 6.14	−0.879	0.387
Disease duration (years)	4.87 ± 3.44	3.72 ± 2.16	−1.074	0.293
Duration of medication (years)	0.5 (0, 4.4)	1.6 (0.1, 3.3)	−0.465	0.642
MDS-UPDRS I	8.92 ± 4.84	8.87 ± 6.09	−0.027	0.979
MDS-UPDRS II	15.85 ± 8.26	11.40 ± 6.62	−1.581	0.126
MDS-UPDRS III	38.54 ± 8.80	31.87 ± 18.44	−1.247	0.226
MDS-UPDRS IV	0 (0, 0)	0 (0, 1.0)	−0.546	0.717
H&Y stage	2.0 (2.0, 2.5)	2.0 (1.0, 3.0)	−1.015	0.310
MMSE	26.00 ± 3.79	27.07 ± 2.58	0.882	0.386
MoCA	21.62 ± 5.55	22.13 ± 3.57	0.284	0.779
PDQ-39	38.69 ± 27.20	33.00 ± 21.31	−0.621	0.540
Baseline LEDD (mg)	319.23 ± 311.49	310.32 ± 311.48	−0.604	0.551
Medication (mg)				
Δlevodopa and benserazide	125.0 (0.281.3)	62.5 (0.187.5)	−0.314	0.753
Δcarbidopa-levodopa	0 (0.0)	0 (0.62.5)	−1.727	0.084
Δentacapone	0 (0.250.0)	0 (0.100.0)	−0.485	0.628
Δpramipexole	0 (0.0.750)	0 (0.0.375)	−0.528	0.597
Δpiribedil	0 (0.50.0)	0 (0.0)	−2.082	0.037*
Δamantadine	0 (0.0)	0 (0.0)	−0.345	0.730
Δselegiline	0 (0.0)	0 (0.0)	−0.414	0.679
Δrasagiline	0 (0.0)	0 (0.0)	−1.074	0.283
Δtrihexyphenidyl	0 (0.0)	0 (0.0)	−1.074	0.283
ΔLEDD (mg)	240.02 ± 214.93	185.68 ± 165.72	−0.760	0.454

Note: Normal data are expressed as means ± standard deviations; Nonnormal data are expressed as median (lower quartile, upper quartile).

MDS-UPDRS: Movement Disorder Society-sponsored Revision of the Unified Parkinson’s Disease Rating Scale; H-Y stage, Hoehn and Yahr stage; MMSE, mini-mental state examination; MoCA, montreal cognitive assessment; PDQ-39, 39-Item Parkinson’s Disease Questionnaire; LEDD, levodopa equivalent daily dose.

*p < 0.05.

### 3.2 Comparison of treatment outcomes between the two groups

**FIGURE 1:**
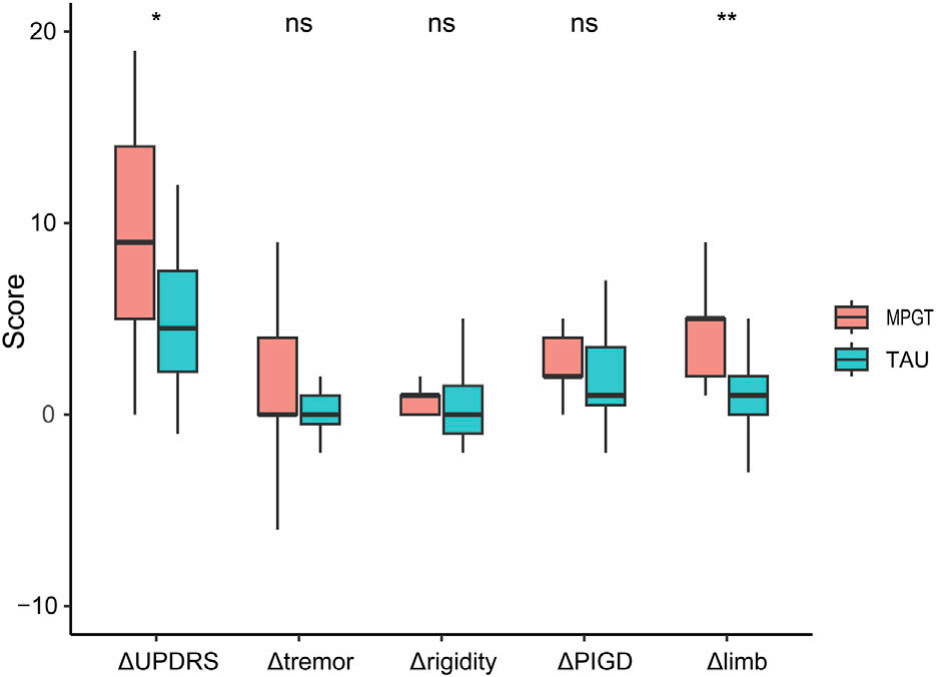
Comparison of UPDRS III score and sub-score reductions between the two groups over 4 weeks. ^*^ *p* < 0.05; ^**^ *p* < 0.01; ns, not significant. Abbreviations: UPDRS III, Part III of the Movement Disorder Society-sponsored Revision of the Unified Parkinson’s Disease Rating Scale; PIGD, postural instability and gait difficulty.

The MPGT group exhibited a significantly greater reduction in the UPDRS III score (9.46 ± 5.47 vs. 2.69 ± 7.95; t = −2.586, *p* < 0.05) and limb scores (4.08 ± 2.66 vs. 1.00 ± 2.75; t = −2.996, *p* < 0.01) from baseline to follow-up compared to the TAU group (Figure 1). After adjusting for the increase in LEDD (OR = −6.22, 95% CI: −11.03–1.42, *p =* 0.011, and OR = −3.08, 95% CI: −5.04–1.12, *p =* 0.002, respectively) or piribedil (OR = −7.50, 95% CI: −12.83–2.16, *p =* 0.006, and OR = −3.06, 95% CI: −5.17–0.96, *p =* 0.004, respectively), the differences remained significant (Supplementary Table S2).

### 3.3 Association between genotypes and treatment outcomes

We further evaluated all the 22 patients who underwent multigenetic pharmacogenomic tests to assess the contribution of each polymorphism to the treatment outcomes. The distribution of all variants complied with Hardy-Weinberg equilibrium. The *DRD2* rs2283265 and rs1076560 polymorphisms were found to be in high linkage disequilibrium with each other (Eryilmaz et al., 2020) (Table 2).

**TABLE 2:** Target genotype distribution (n = 22).

SNP	Gene	Major/Minor allele	MAF^a^	Genotype	Frequencies, n (%)	HWE, *p*-value
rs4984241	*CA12*	A/G	0.41	AA	8 (36.4)	0.071
				AG	5 (22.7)	
				GG	9 (40.9)	
rs4680	*COMT*	G/A	0.28	GG	11 (50.0)	0.794
				GA	10 (45.5)	
				AA	1 (4.5)	
rs1799732	*DRD2*	G/-	0.1	GG	17 (77.3)	0.856
				G/-	5 (22.7)	
				−/−	0 (0)	
rs1076560	*DRD2*	C/A	0.42	CC	4 (18.2)	0.441
				AC	14 (63.6)	
				AA	4 (18.2)	
rs2283265	*DRD2*	C/A	0.25	CC	4 (18.2)	0.441
				AC	14 (63.6)	
				AA	4 (18.2)	
rs6280	*DRD3*	T/C	0.31	TT	8 (36.4)	0.697
				CT	12 (54.5)	
				CC	2 (9.1)	
rs76126170	*DRD3*	C/T	0.13	CC	19 (86.4)	0.950
				CT	3 (13.6)	
rs9817063	*DRD3*	T/C	0.45	TT	7 (31.8)	0.998
				CT	11 (50.0)	
				CC	4 (18.2)	
rs9868039	*DRD3*	G/A	0.37	GG	7 (31.8)	0.441
				AG	8 (36.4)	
				AA	7 (31.8)	
rs622342	*SLC22A1*	A/C	0.14	AA	18 (81.8)	0.865
				AC	4 (18.2)	
rs4704559	*HOMER1*	A/G	0.09	AA	18 (81.8)	0.199
				AG	3 (13.6)	
				GG	1 (4.5)	
rs3832043	*UGT1A9*	(T)10/(T)9	0.03	2(T)10	6 (27.3)	0.224
				(T)10/(T)9	13 (59.1)	
				2(T)9	3 (13.6)	

^a^ Based on the allele frequency of East Asian from NCBI SNP database.

SNP, single nucleotide polymorphism; MAF, minor allele frequency; HWE, Hardy–Weinberg equilibrium.

For *CA12* rs4984241, patients with the AA genotype (n = 8) and those with the AG or GG genotype (n = 14) differed significantly in age (*p* < 0.01). No significant differences in baseline characteristics were observed between genotype groups for *COMT* rs4680, *DRD2* rs1076560/rs2283265, and *SLC22A1* rs622342 (Table 3). Due to the limited number of subjects, it was necessary to exclude *DRD2* rs1799732 and *DRD3* rs76126170 from assessing the relationship between genotype and UPDRS motor scale outcomes in dominant models. Additionally, *COMT* rs4680, *DRD3* rs6280, *DRD2* rs1799732, *DRD3* rs76126170, *HOMER1* rs4704559, and *UGT1A9* rs3832043 were excluded from recessive and additive models.

**TABLE 3:** Baseline characteristics of patients in each genotype group under the dominant model.

Genotype	rs4984241			rs4680			rs1076560 and rs2283265			rs622342		
	AA (n = 8)	AG + GG (n = 14)	*p*-value	GG (n = 11)	GA + AA (n = 11)	*p*-value	CC (n = 4)	AC + AA (n = 18)	*p*-value	AA (n = 18)	AC (n = 4)	*p*-value
Gender (Male/Female)	6/2	8/6	0.649	8/3	6/5	0.659	3/1	11/7	1.000	11/7	3/1	1.000
Age (years)	62.88 ± 6.27	71.07 ± 6.10	0.007**	68.91 ± 7.96	67.27 ± 6.72	0.608	63.75 ± 8.77	69.06 ± 6.76	0.191	67.28 ± 7.40	71.75 ± 5.85	0.274
Disease duration (years)	4.91 ± 3.81	4.66 ± 2.35	0.851	5.12 ± 3.49	4.39 ± 2.21	0.566	4.13 ± 2.43	4.89 ± 3.01	0.640	4.82 ± 2.88	4.45 ± 3.27	0.821
Duration of medication (years)	0.8 (0.2, 2.5)	2.2 (0, 5.3)	0.654	1.0 (0, 6.5)	2.3 (0, 3.5)	0.842	1.7 (0.1, 3.4)	1.3 (0, 5.3)	0.830	1.3 (0, 3.7)	2.3 (0.3, 5.8)	0.667
MDS-UPDRS I	6.50 ± 4.38	11.36 ± 5.83	0.055	9.27 ± 4.84	9.91 ± 6.79	0.803	6.00 ± 3.65	10.39 ± 5.91	0.174	9.28 ± 5.93	11.00 ± 5.48	0.601
MDS-UPDRS II	12.88 ± 7.22	14.86 ± 7.90	0.566	16.55 ± 8.70	11.73 ± 5.59	0.138	9.75 ± 5.06	15.11 ± 7.77	0.206	13.11 ± 5.48	18.75 ± 12.79	0.183
MDS-UPDRS III	34.25 ± 12.33	35.14 ± 15.54	0.891	38.82 ± 8.73	30.82 ± 17.59	0.192	29.50 ± 17.14	36.00 ± 13.69	0.419	35.83 ± 15.43	30.25 ± 4.35	0.489
MDS-UPDRS IV	0 (0, 0)	0 (0, 5.0)	0.207	0 (0, 1.0)	0 (0, 4.0)	1.000	0 (0, 3.0)	0 (0, 2.0)	0.786	0 (0, 1.8)	0 (0, 8.3)	0.871
H&Y stage	2.0 (2.0, 2.5)	2.0 (1.9, 2.6)	0.914	2.0 (2.0, 2.5)	2.0 (1.0, 3.0)	0.238	2.0 (1.3, 2.0)	2.0 (2.0, 2.6)	0.163	2.0 (1.9, 2.6)	2.0 (2.0, 2.4)	0.928
MMSE	27.88 ± 2.10	25.71 ± 3.60	0.139	27.00 ± 3.10	26.00 ± 3.49	0.486	27.50 ± 2.65	26.28 ± 3.41	0.511	26.330 ± 3.52	27.25 ± 1.89	0.623
MoCA	23.57 ± 5.19	20.08 ± 4.92	0.154	23.27 ± 4.65	18.89 ± 4.96	0.057	21.67 ± 7.10	21.24 ± 5.04	0.898	20.81 ± 5.50	23.25 ± 3.40	0.414
PDQ-39	33.00 ± 25.69	37.71 ± 24.27	0.672	37.18 ± 30.41	34.82 ± 17.63	0.826	28.75 ± 13.89	37.61 ± 26.07	0.523	31.67 ± 17.70	55.50 ± 41.64	0.338
ΔLEDD (mg)	229.75 ± 251.77	154.82 ± 128.41	0.362	243.89 ± 223.06	120.25 ± 102.47	0.110	187.50 ± 145.06	180.86 ± 191.49	0.949	149.01 ± 112.80	330.81 ± 347.46	0.374

**p < 0.01.

In the dominant models, the ΔUPDRS III was significantly higher in patients carrying the *CA12* AA genotype than those with the AG or GG genotype, after adjusting for age and ΔLEDD (AA vs. AG + GG, OR = 10.81, 95% CI: 3.65–17.97, *p =* 0.003). Genotypes for rs4680 were associated with improvements in the PIGD sub-score both before (*p =* 0.028) and after adjusting for ΔLEDD (GG vs. GA + AA, OR = 3.01, 95% CI: 0.63–5.39, *p =* 0.013). In addition, improvement in rigidity scores was greater in patients with CC genotype of *DRD2* rs1076560/rs2283265 than in those with AC or AA genotype, after adjusting for ΔLEDD (CC vs. AC + AA, OR = 1.86, 95% CI: 0.09–3.62, *p =* 0.039). Tremor sub-scores decreased more in patients with the *SLC22A1* rs622342 AA genotype after adjusting for ΔLEDD (AA vs. AC, OR = 3.90, 95% CI = 0.13–7.67, *p =* 0.043) (Table 4). No significant associations were found between medication adjustment response and polymorphisms in the recessive and additive models (Tables 5, 6).

**TABLE 4:** Effect of genotypes on the improvement of UPDRS motor scores after medication adjustment in dominant models (n = 22).

Gene	SNP	Genotype	Δ UPDRS III	Δ tremor	Δ rigidity	Δ PIGD	Δ limb
*CA12*	rs4984241	AA (n = 8)	8.25 ± 6.65	1.25 ± 4.95	1.13 ± 0.991	4.25 ± 3.77	3.88 ± 3.14
		AG+GG (n = 14)	3.88 ± 9.01	0.50 ± 2.85	0.07 ± 2.40	1.57 ± 2.28	2.07 ± 3.32
		*t*	1.194	0.455	1.175	2.092	1.250
		*p*-value	0.246	0.654	0.254	0.049*	0.226
		*p* ^a^-value	0.003**	0.776	0.708	0.085	0.003**
*COMT*	rs4680	GG (n = 11)	7.39 ± 5.33	1.27 ± 3.82	0.91 ± 1.45	4.00 ± 3.52	3.18 ± 2.44
		GA+AA (n = 11)	3.55 ± 10.47	0.27 ± 3.58	0.00 ± 2.49	1.09 ± 1.81	2.27 ± 4.05
		*t/t*’	1.085	0.663	1.047	2.436	0.637
		*p*-value	0.291	0.534	0.308	0.028*	0.531
		*p* ^b^-value	0.439	0.746	0.694	0.013*	0.537
*DRD2*	rs1076560/rs2283265	CC (n = 4)	9.75 ± 8.06	0.50 ± 5.45	2.00 ± 1.83	2.00 ± 1.41	4.50 ± 3.32
		AC+AA (n = 18)	4.52 ± 8.32	0.83 ± 3.35	0.11 ± 1.97	2.67 ± 3.40	2.33 ± 3.25
		*t*	1.143	−0.161	1.756	−0.379	1.201
		*p*-value	0.267	0.873	0.094	0.708	0.244
		*p* ^b^ -value	0.222	0.850	0.039*	0.682	0.208
*DRD3*	rs6280	TT (n = 8)	7.38 ± 5.83	0.50 ± 4.31	0.50 ± 1.41	2.00 ± 3.02	3.38 ± 2.62
		CT + CC (n = 14)	4.38 ± 9.52	0.93 ± 3.39	0.43 ± 2.38	2.86 ± 3.23	2.36 ± 3.67
		*t*	0.803	−0.259	0.077	−0.612	0.688
		*p*-value	0.431	0.798	0.939	0.547	0.500
		*p* ^b^ -value	0.283	0.900	0.658	0.184	0.573
*DRD3*	rs9817063	TT (n = 7)	8.00 ± 4.12	0.00 ± 4.32	0.14 ± 1.77	2.86 ± 2.80	3.71 ± 2.36
		CT+CC (n = 15)	4.29 ± 9.62	1.13 ± 3.40	0.60 ± 2.20	2.40 ± 3.33	2.27 ± 3.63
		*t*	0.971	−0.669	−0.480	0.314	0.957
		*p*-value	0.343	0.511	0.636	0.757	0.350
		*p* ^b^ -value	0.157	0.632	0.997	0.626	0.270
*DRD3*	rs9868039	GG (n = 7)	6.14 ± 7.67	−0.29 ± 0.95	1.43 ± 1.72	2.29 ± 1.89	2.57 ± 2.94
		AG+AA (n = 15)	5.15 ± 8.88	1.27 ± 4.33	0.00 ± 2.07	2.67 ± 3.60	2.80 ± 3.55
		*t/t’*	0.253	−1.321	1.583	−0.261	−0.148
		*p*-value	0.803	0.204	0.129	0.796	0.884
		*p* ^b^ -value	0.776	0.091	0.415	0.549	0.768
*HOMER1*	rs4704559	AA (n = 18)	5.13 ± 8.97	0.56 ± 3.88	0.44 ± 2.26	2.72 ± 3.43	2.78 ± 3.52
		AG + GG (n = 4)	7.00 ± 5.23	1.75 ± 2.50	0.50 ± 0.58	1.75 ± 0.50	2.50 ± 2.38
		*t*	−0.398	−0.583	−0.048	0.556	0.149
		*p*-value	0.695	0.567	0.962	0.585	0.883
		*p* ^b^ -value	0.593	0.473	0.802	0.589	0.892
*SLC22A1*	rs622342	AA (n = 18)	4.91 ± 8.96	1.22 ± 3.77	0.11 ± 1.78	2.44 ± 3.37	2.72 ± 3.61
		AC (n = 4)	8.00 ± 4.69	−1.25 ± 2.50	2.00 ± 2.71	3.00 ± 1.83	2.75 ± 1.50
		*t*	−0.662	1.241	−1.756	−0.316	−0.015
		*p*-value	0.516	0.229	0.094	0.755	0.988
		*p* ^b^ -value	0.807	0.043*	0.274	0.917	0.926
*UGT1A9*	rs3832043	A(T)10AT (n = 6)	6.00 ± 2.19	0.67 ± 3.56	0.83 ± 1.94	2.00 ± 1.67	1.83 ± 0.75
		A(T)10AT/A(T)9AT+A(T)9AT (n = 16)	5.27 ± 9.78	0.81 ± 3.80	0.31 ± 2.12	2.751 ± 3.53	3.06 ± 3.82
		*t/t’*	0.281	−0.081	0.524	−0.494	−0.771
		*p*-value	0.782	0.936	0.606	0.627	0.450
		*p* ^b^ -value	0.872	0.683	0.945	0.466	0.354

^a^ Adjusted for increased levodopa equivalent daily dose (ΔLEDD) and age.

^b^ Adjusted for increased levodopa equivalent daily dose (ΔLEDD).

*p < 0.05; **p < 0.01.

**TABLE 5:** Effect of genotypes on the improvement of UPDRS motor scores after medication adjustment in recessive models (n = 22).

Gene	SNP	Genotype	Δ UPDRS III	Δ tremor	Δ rigidity	Δ PIGD	Δ limb
*CA12*	rs4984241	GG (n = 9)	4.56 ± 11.23	0.78 ± 2.77	−0.11 ± 2.47	1.33 ± 1.80	2.11 ± 4.14
		AG+AA (n = 13)	6.10 ± 6.07	0.77 ± 4.27	0.85 ± 1.68	3.38 ± 3.60	3.15 ± 2.67
		*t*	−0.418	0.005	−1.086	−1.572	−0.721
		*p*-value	0.680	0.996	0.290	0.132	0.479
		*p* ^a^-value	0.706	0.942	0.260	0.105	0.458
*DRD2*	rs1076560/rs2283265	AA (n = 4)	−1.17 ± 12.40	0.25 ± 2.87	−0.25 ± 3.40	1.75 ± 3.86	0.25 ± 4.57
		AC+CC (n = 18)	6.94 ± 6.80	0.89 ± 3.86	0.61 ± 1.72	2.72 ± 3.03	3.28 ± 2.82
		*t*	−1.861	−0.310	−0.756	−0.556	−1.739
		*p*-value	0.078	0.760	0.459	0.585	0.097
		*p* ^a^-value	0.067	0.852	0.580	0.612	0.071
*DRD3*	rs9817063	CC (n = 4)	6.50 ± 10.54	−0.75 ± 0.96	2.00 ± 2.16	2.50 ± 2.65	2.25 ± 3.78
		CT+TT (n = 18)	5.24 ± 8.13	1.11 ± 3.95	0.11 ± 1.91	2.56 ± 3.28	3.83 ± 3.29
		*t*	0.267	−0.919	1.756	−0.032	−0.313
		*p*-value	0.792	0.369	0.094	0.975	0.757
		*p* ^a^-value	0.671	0.051	0.429	0.678	0.582
*DRD3*	rs9868039	AA (n = 7)	8.00 ± 4.12	0.00 ± 4.32	0.14 ± 1.77	2.86 ± 2.80	3.71 ± 2.36
		AG+GG (n = 15)	4.29 ± 9.62	1.13 ± 3.40	0.60 ± 2.20	2.40 ± 3.33	2.27 ± 3.63
		*t*	0.971	−0.669	−0.480	0.314	0.957
		*p*-value	0.343	0.511	0.636	0.757	0.350
		*p* ^a^-value	0.157	0.632	0.997	0.626	0.270

^a^ Adjusted for increased levodopa equivalent daily dose (ΔLEDD) and age.

*p < 0.05; **p < 0.01.

**TABLE 6:** Effect of genotypes on the improvement of UPDRS motor scores after medication adjustment in additive models (n = 22).

Gene	SNP	Genotype	Δ UPDRS III	Δ tremor	Δ rigidity	Δ PIGD	Δ limb
*CA12*	rs4984241	AA (n = 8)	8.25 ± 6.65	1.25 ± 4.95	1.13 ± 0.99	4.25 ± 3.77	3.88 ± 3.14
		AG (n = 5)	2.66 ± 2.99	0.00 ± 3.24	0.40 ± 2.51	2.00 ± 3.16	2.00 ± 1.23
		GG (n = 9)	4.56 ± 11.23	0.78 ± 2.77	−0.11 ± 2.47	1.33 ± 1.80	2.11 ± 4.14
		*F*	0.764	0.166	0.762	2.179	0.745
		*p*-value	0.480	0.848	0.481	0.141	0.488
		*p* ^a^-value	0.541	0.915	0.515	0.096	0.437
*DRD2*	rs1076560/rs2283265	CC (n = 4)	9.75 ± 8.06	0.50 ± 5.45	2.00 ± 1.83	2.00 ± 1.41	4.50 ± 3.32
		AC (n = 14)	6.14 ± 6.50	1.00 ± 3.55	0.21 ± 1.53	2.93 ± 3.36	2.93 ± 2.70
		AA (n = 4)	−1.17 ± 12.40	0.25 ± 2.87	−0.25 ± 3.40	1.75 ± 3.86	0.25 ± 4.57
		*F*	2.018	0.072	1.562	0.278	1.878
		*p*-value	0.160	0.931	0.236	0.761	0.180
		*p* ^a^-value	0.117	0.956	0.118	0.758	0.118
*DRD3*	rs9817063	TT (n = 7)	8.00 ± 4.12	0.00 ± 4.32	0.14 ± 1.77	2.86 ± 2.80	3.71 ± 2.36
		CT (n = 11)	3.48 ± 9.67	1.82 ± 3.74	0.09 ± 2.07	2.36 ± 3.67	2.27 ± 3.77
		CC (n = 4)	6.50 ± 10.54	−0.75 ± 0.96	2.00 ± 2.16	2.50 ± 2.65	2.25 ± 3.78
		*F*	0.642	0.951	1.466	0.049	0.435
		*p*-value	0.537	0.404	0.256	0.952	0.653
		*p* ^a^-value	0.366	0.079	0.718	0.846	0.518
*DRD3*	rs9868039	GG (n = 7)	6.14 ± 7.67	−0.29 ± 0.95	1.43 ± 1.72	2.29 ± 1.89	2.57 ± 2.94
		AG (n = 8)	2.66 ± 11.32	2.38 ± 4.31	−0.12 ± 2.42	2.50 ± 4.38	2.00 ± 4.34
		AA (n = 7)	8.00 ± 4.12	0.00 ± 4.32	0.14 ± 1.77	2.86 ± 2.80	3.71 ± 2.36
		*F*	0.781	1.251	1.228	0.055	0.491
		*p*-value	0.472	0.309	0.315	0.947	0.620
		*p* ^a^-value	0.348	0.080	0.668	0.807	0.536

^a^ Adjusted for increased levodopa equivalent daily dose (ΔLEDD) and age.

*p < 0.05; **p < 0.01.

## 4 Discussion

To our knowledge, this is the first real-world longitudinal, perspective cohort study to evaluate the therapeutic efficacy of MPGT in Chinese patients with PD. We found that patients in the MPGT group achieved greater improvements in motor symptoms, particularly in limb bradykinesia. Our results also indicated that patients carrying the *CA12* AA genotype responded better to adjustments of anti-parkinsonian medications. Compared to other allele carriers, GG homozygotes for *COMT* and CC homozygotes for *DRD2* showed greater improvements in PIGD and rigidity, respectively. Additionally, the *SLC22A1* A > C polymorphism was associated with poorer responsiveness in tremor.

As genome-guided therapeutics show promise in personalized dosing and medication selection, efforts have been made to define the effects of gene polymorphisms on therapeutic response and side effects of dopaminergic therapy. However, there are still no clinical guidelines for using pharmacogenomics in the treatment of PD. Data on Parkinson’s disease in PharmGKB, a pharmacogenomics database, are sparse, with only nine clinical annotations, most of which are supported by a relatively low level of evidence. The multigenetic pharmacogenomic testing in our study included all genetic variants mentioned in PharmGKB clinical annotations. Genetic polymorphisms of metabolizing enzymes, such as *CYP1A2* for the response to ropinirole and rasagiline, and *CYP3A4* for the response to istradefylline, were also detected (Agundez et al., 2013). Other genetic variants detected in this study were related to treatment responses or adverse effects, including *DRD3* rs76126170, rs9817063, and rs9868039 for responses to piribedil (Zhang et al., 2021), *CA12* rs2306719, rs4984241 and *HLA-A* for adverse effects to zonisamide (Mirza et al., 2011; Kaniwa et al., 2013), *APOE* for adverse effects to DRT (de la Fuente-Fernandez et al., 1999) or trihexyphenidyl (Pomara et al., 2008), and UGT1A9*22 for adverse effects to entacapone or tolcapone (Yamanaka et al., 2004). In our study, we found that the dosage of piribedil increased more in the MPGT group, indicating that pharmacogenomic reports influenced medication selection. However, there was no significant difference in the ΔLEDD between the two groups. Patients in MPGT group responded better to anti-parkinsonian drugs, as evidenced by a greater reduction in UPDRS III score and its limbs sub-scores. The improvement in motor symptoms might be explained by the interaction of different medications.

In contrast to diseases typically treated with monotherapy, the initial treatment of PD almost always involves levodopa preparations, with or without dopamine agonists and monoamine oxidase-B (MAO-B) inhibitors (Armstrong and Okun, 2020). As the disease progresses, catechol-O-methyltransferase (COMT) inhibitors are added to extend the benefits of levodopa. Amantadine and trihexyphenidyl are prescribed only in specific cases. Other classes of drugs were not available in our clinical practice. Therefore, personalized therapy primarily depended on selecting the appropriate dopamine agonist and determining when to add entacapone. Piribedil was more frequently recommended in our reports because three SNPs (*DRD3* rs76126170, rs9817063 and rs9868039) were associated with treatment responses (Zhang et al., 2021), while no SNPs were linked to adverse effects in the previous literature. A total of four patients in the MPGT group were prescribed piribedil based on pharmacogenomic test recommendations. However, one of them discontinued the medication due to nausea and vomiting, highlighting the importance of incorporating SNPs related to both treatment response and tolerability into MPGT algorithms for accurate recommendations. Another case is levodopa. While there is robust evidence for the pharmacogenomics of levodopa’s side effects, only one polymorphism, *SLC6A3* rs3836790, has been associated with the motor response to levodopa (Moreau et al., 2015). However, this correlation was not found in our study, in accordance with another study from China (Li et al., 2020). As a result, levodopa was not recommended as the optimal medication for any of the patients, which contrasts with common clinical practice. In conclusion, new genes and loci associated with the efficacy and safety of commonly used drugs need to be further explored.

Although all the tested SNPs have been reported in previous literature, their effects on the chronic administration of multiple anti-parkinsonian drugs have not been verified. Therefore, we further evaluated the effect of each genotype on treatment outcomes in all tested patients. Unexpectedly, an association was observed between *CA12* genotypes and motor response. CA XII, one of the major isoforms in human kidneys, plays an essential role in the reabsorption of ultrafiltered HCO3- by the proximal tubule and in urinary acidification by the distal tubule (Purkerson and Schwartz, 2007). The AA genotype of rs4984241 was associated with significantly lower serum bicarbonate levels in patients on topiramate or zonisamide (Mirza et al., 2011). A previous report showed that amantadine uptake was stimulated by bicarbonate in the proximal tubules (Goralski et al., 2002). We speculated that this *CA12* polymorphism enhances the uptake of certain anti-parkinsonian drugs, such as amantadine. However, the role of *CA12* rs4984241 as a possible biomarker for anti-parkinsonian treatment response remains unclear and further investigations are necessary to confirm its role.

Another polymorphism, rs4680 (G > A), which is linked to low COMT enzyme activity, was associated with higher levodopa dosages, suggesting a better response to chronic levodopa administration (Bialecka et al., 2004; Bialecka et al., 2008; Cheshire et al., 2014). However, the high-activity genotype (GG) was associated with an increased response to entacapone (Corvol et al., 2011). In our study, the PIGD sub-score improved more in patients carrying the GG genotype after medication adjustment. This effect could not be attributed to any specific medication, as majority of patients were on multiple anti-parkinsonian drugs.

Furthermore, our results confirmed that *DRD2* rs1076560/rs2283265 is related to the motor response to anti-parkinsonian treatment. It has been reported that CC homozygotes for rs1076560 and rs2283265 in *DRD2* showed an earlier and greater improvement in PD symptoms when treated with rasagiline compared to A allele carriers (Masellis et al., 2016). It was also noted that A allele carriers had worse gait dysfunction compared to C allele homozygotes who were treated with various anti-parkinsonian medications (Miller et al., 2018). Consistent with these findings, the CC genotype of these polymorphisms was associated with larger improvement in rigidity following adjusted medication in our study.

Additionally, our study showed a lower response in tremor to medication adjustments in patients carrying the C allele of *SLC22A1* rs622342. Organic cation transporter 1 (OCT 1), encoded by the *SLC22A1* gene, is involved in the transport of metformin and certain anti-parkinsonian drugs, such as amantadine, pramipexole, and, possibly levodopa (Gomes et al., 1997; Goralski et al., 2002; Jonker and Schinkel, 2004; Ishiguro et al., 2005; Okura et al., 2007). The minor C allele of rs622342 is likely associated with reduced OCT1 transporter function. In a population-based cohort study, the minor C allele was associated with higher prescribed doses of anti-parkinsonian drugs (Becker et al., 2011), suggesting a lower response to one or more of these medications.

The analysis of other SNPs, such as rs3836790 in *SLC6A3* and various polymorphisms in *DRD3*, which have been previously reported to be associated with the effects of levodopa (Moreau et al., 2015), pramipexole (Liu et al., 2009; Xu et al., 2017) or piribedil (Zhang et al., 2021), did not reveal any significant associations with repeated administrations of multiple drugs in our study.

The highlight of this study is its prospective, longitudinal design. Our study demonstrated that combining clinical experience with MPGT improved drug response in Chinese PD patients. Furthermore, all patients included in the study were carefully assessed to rule out atypical Parkinson’s syndrome through follow-up and dopaminergic imaging. We also excluded those with prominent non-motor symptoms and monogenic forms of PD, where applicable, to reduce disease heterogeneity.

Our study had several limitations, primarily due to the relatively small sample size. Only one advanced-stage PD patient developed dyskinesias, while another patient developed gastrointestinal symptoms after piribedil treatment. Given the limited number of cases, a meaningful comparison of adverse effects between the two groups and an analysis of their association with different genotypes were difficult. We recognize the importance of this aspect and plan to address it in future studies with a larger sample size. Secondly, since this was an observational study, patients were not randomized into groups, and the raters were not blinded to the study group. A double-blinded, randomized clinical trial is required to validate these finding. Thirdly, the algorithm should account for ethnic differences and specific SNPs that differently affect responses to acute challenge tests or chronic medication administration. To fully personalize PD treatment, considerable work remains. More validated gene variants related to pharmacokinetics and pharmacodynamics must be incorporated.

In summary, our study demonstrated that pharmacogenomics-guided treatment may improve motor symptoms in PD patients. However, large-scale, randomized controlled trials are needed to validate these findings.

## Data Availability

The original contributions presented in the study are included in the article/Supplementary Material, further inquiries can be directed to the corresponding authors.
