# Supplementary material for: Multigenetic pharmacogenomics–guided treatment shows greater improvements on motor symptoms compared to usual therapy in Parkinson’s disease: a small real-word prospective cohort study
Source: Front Pharmacol. 2025 Mar 25;16:1502379. doi: 10.3389/fphar.2025.1502379 (PMC11975922; doi:10.3389/fphar.2025.1502379)
Supplement: Supplementary file 1 [file Table1.docx]

Supplementary Material

# Supplementary Table

## Supplementary Table 1 Genotype distribution of participants.

| Gene (SNP) | Genotype / Metabolizer | Patients, No. (%) | | |
| --- | --- | --- | --- | --- |
|  |  | MPGT (n=14) | TAU (n=8) | Total (n=22) |
| *CYP1A2* | UM | 4 (28.6) | 2 (25.0) | 6 (27.3) |
|  | NM | 10 (71.4) | 6 (75.0) | 16 (72.7) |
| *CYP3A4* | EM | 14 (100) | 8 (100) | 22 (100) |
| *CA12*  (rs2306719) | TT | 14 (100) | 8 (100) | 22 (100) |
| *CA12*  (rs4984241) | AA | 7 (50.0) | 1 (12.5) | 10 36.4) |
|  | AG | 1 (7.1) | 4 (50.0) | 5 (22.7) |
|  | GG | 6 (42.9) | 3 (37.5) | 9 (40.9) |
| *COMT*  (rs4680) | GG | 10 (71.4) | 1 (12.5) | 11 (50.0) |
|  | GA | 4 (28.6) | 6 (75.0) | 10 (45.5) |
|  | AA | 0 (0) | 1 (12.5) | 1 (4.5) |
| *DRD2*  (rs1076560) | CC | 2 (14.3) | 2 (25.0) | 4 (18.2) |
|  | AC | 10 (71.4) | 4 (50.0) | 14 (63.6) |
|  | AA | 2 (14.3) | 2 (25.0) | 4 (18.2) |
| *DRD2*  (rs1799732) | GG | 10 (71.4) | 7 (87.5) | 17 (77.3) |
|  | G/- | 4 (28.6) | 1 (12.5) | 5 (22.7) |
| *DRD2*  (rs2283265) | CC | 2 (14.3) | 2 (25.0) | 4 (18.2) |
|  | AC | 10 (71.4) | 4 (50.0) | 14 (63.6) |
|  | AA | 2 (14.3) | 2 (25.0) | 4 (18.2) |
| *DRD3*  (rs6280) | TT | 4 (28.6) | 4 (50.0) | 8 (36.4) |
|  | CT | 10 (71.4) | 2 (25.0) | 12 (54.5) |
|  | CC | 0 (0) | 2 (25.0) | 2 (9.1) |
| *DRD3*  (rs76126170) | CC | 11 (78.6) | 8 (100) | 19 (86.4) |
|  | CT | 3 (21.4) | 0 (0) | 3 (13.6) |
| *DRD3*  (rs9817063) | TT | 4 (28.6) | 3 (37.5) | 7 (31.8) |
|  | CT | 7 (50.0) | 4 (50.0) | 11 (50.0) |
|  | CC | 3 (21.4) | 1 (12.5) | 4 (18.2) |
| *DRD3*  (rs9868039) | GG | 6 (42.9) | 1 (12.5) | 7 (31.8) |
|  | AG | 4 (28.6) | 4 (50.0) | 8 (36.4) |
|  | AA | 4 (28.6) | 3 (37.5) | 7 (31.8) |
| *APOE* | ε2/ε3 | 1 (7.1) | 2 (25.0) | 3 (13.6) |
|  | ε3/ε3 | 6 (42.9) | 4 (50.0) | 10 (45.5) |
|  | ε3/ε4 | 7 (50.0) | 2 (25.0) | 9 (40.9) |
| *HLA-A* | NEG | 13 (92.9) | 8 (100) | 21 (95.5) |
|  | POS | 1 (7.1) | 0 (0) | 1 (4.5) |
| *HOMER1* (rs4704559) | AA | 12 (85.7) | 6 (75.0) | 21 (81.8) |
|  | AG | 2 (14.3) | 1 (12.5) | 3 (13.6) |
|  | GG | 0 (0) | 1 (12.5) | 1 (4.5) |
| *SLC22A1*  (rs622342) | AA | 11 (78.6) | 7 (87.5) | 18 (81.8) |
|  | AC | 3 (21.4) | 1 (12.5) | 4 (18.2) |
| *SLC6A3*  (rs3836790) | Ins | 14 (100) | 8 (100) | 22 (100) |
| *UGT1A9*  (rs3832043) | A(T)10AT | 4 (28.6) | 2 (25.0) | 6 (27.3) |
|  | A(T)10AT/A(T)9AT | 8 (57.1) | 5 (62.5) | 13 (59.1) |
|  | A(T)9AT | 2 (14.3) | 1 (12.5) | 3 (13.6) |

CYP1A2, Cytochrome P450 Family 1 Subfamily A Member 2; CYP3A4, Cytochrome P450 Family 3 Subfamily A Member 4; CA12, Carbonic Anhydrase 12; COMT, Catechol-O-methyltransferase; DRD2, Dopamine Receptor D2; DRD3, Dopamine Receptor D3; APOE, Apolipoprotein E; HLA-A, major histocompatibility complex, class I, A; HOMER1, Homer Scaffold Protein 1; SLC22A1, Solute Carrier Family 22 Member 1; UGT1A9, UDP Gglucuronosyltransferase Family 1 Member A9; IM, intermediate metabolizer; NM, normal metabolizer; PM, poor metabolizer; UM, ultrarapid metabolizer; EM, Extensive Metabolizer.

Supplementary Table 2 Comparison of UPDRS Ⅲ score and sub-score reductions between the two groups over 4 weeks.

|  | MPGT (n=13) | TAU (n=15) | *t* | *p*-value | *p*^a^-value | *p^b^*-value |
| --- | --- | --- | --- | --- | --- | --- |
| ΔUPDRS Ⅲ | 9.46 ± 5.47 | 2.69 ± 7.95 | -2.586 | 0.016^*^ | 0.011^*^ | 0.006^*^ |
| Δtremor | 1.69 ± 3.99 | 0.20 ± 2.48 | -1.206 | 0.239 | 0.267 | 0.282 |
| Δrigidity | 0.92 ± 1.50 | 0.33 ± 2.44 | -0.756 | 0.457 | 0.673 | 0.469 |
| ΔPIGD | 3.46 ± 3.15 | 1.87 ± 2.56 | -1.478 | 0.152 | 0.142 | 0.646 |
| Δlimb | 4.08 ± 2.66 | 1.00 ± 2.75 | -2.996 | 0.006^**^ | 0.002^**^ | 0.004^**^ |

Note: Normal data are expressed as means ± standard deviations; Nonnormal data are expressed as median (lower quartile, upper quartile).

UPDRS Ⅲ, Part III of the Movement Disorder Society-sponsored Revision of the Unified Parkinson’s Disease Rating Scale; PIGD, postural instability and gait difficulty.

^a^ Adjusted for increased levodopa equivalent daily dose (ΔLEDD).

^b^ Adjusted for increased piribedil (Δ piribedil).

^*^*p <* 0.05; ^**^*p <* 0.01.
